# Editorial: Artificial intelligence in radiology and radiation oncology

**DOI:** 10.3389/fradi.2025.1657119

**Published:** 2025-07-23

**Authors:** Curtise K. C. Ng, Vincent W. S. Leung

**Affiliations:** ^1^ Curtin Medical School, Curtin University, Perth, WA, Australia; ^2^ Curtin Medical Research Institute (Curtin MRI), Faculty of Health Sciences, Curtin University, Perth, WA, Australia; ^3^ Department of Health Technology and Informatics, Faculty of Health and Social Sciences, The Hong Kong Polytechnic University, Hong Kong, Hong Kong SAR, China

**Keywords:** artificial intelligence, computer-aided detection, deep learning, image synthesis, medical imaging, radiation oncology, radiology, radiomics

**Editorial on the Research Topic**
Artificial intelligence in radiology and radiation oncology

Use of artificial intelligence (AI) has become popular in radiology and radiation oncology practices. Various commercial products such as image quality enhancement and computer-aided detection and diagnosis (CAD) tools in radiology, and auto-segmentation software in radiation therapy treatment planning are available nowadays (1–5). To promote ongoing development of AI in radiology and radiation oncology, this Research Topic provides a platform to facilitate the sharing of respective AI research findings and promote evidence-based practice. This Research Topic includes four distinct studies covering both machine learning [least absolute shrinkage and selection operator (LASSO)] and deep learning (DL) techniques [convolutional neural network (CNN), generative adversarial network (GAN) and Vision Transformers (ViT)] for image synthesis, registration, segmentation, classification, CAD and radiomics. We would like to thank all authors for their contributions.

The contribution by Lerch et al. demonstrates that their GAN model, *DreamOn* is able to synthesize images for training the ResNet-18 model for breast ultrasound image classification, resulting in more robust model performance. Their study findings are consistent with outcomes of a previous systematic review that GAN is an effective data augmentation strategy (6). For researchers who are interested in using their *DreamOn* model for data augmentation, the model can be obtained from https://github.com/lucle4/DreamOn.

For Dumbrique et al.'s contribution, a novel DL model based on fully CNNs (FCNNs) and ViTs is developed for CAD of pneumothorax. Their model outperforms those of previous studies by Abdella et al. (7), Hongyu et al. (8), Jakhar et al. (9), Malhotra et al. (10) and Mostayed et al. (11). This paves the way for implementation of their model in clinical practice.

Guo et al.'s study shows that their machine learning based radiomics model is effective for luminal and non-luminal breast cancer differentiation based on T2-weighted magnetic resonance images with the highest area under the receiver operating characteristic curve value of 0.873. Although this result is encouraging, manual segmentation of tumors is employed in their study. This subsequently affects its generalizability to certain extent (12).

The last contribution covered in this Research Topic is about a research platform, SenseCare developed by Wang et al. The SenseCare allows multi-institution access to a range of DL models for image registration, segmentation, classification and CAD in radiology and radiation oncology. These tools support translational research projects across different clinical domains.

It is noted that the AI applications in radiology and radiation oncology are broad. In this Research Topic, several representations of these applications are demonstrated. However, other uses such as automated structured reporting, clinical decision support, image quality enhancement, reconstruction and translation as well as optimization of examination/treatment scheduling are not included (13). Radiology and radiation oncology are leading specialties in the adoption of AI applications in healthcare. As of 2021, about 70% of the 343 United States Food and Drug Administration approved AI products were developed for these two specialties (14). According to the European Network for the Assessment of Imaging in Medicine (EuroAIM)/European Society of Medical Imaging Informatics (EuSoMII) 2024 survey with 572 responses from the European Society of Radiology (ESR) members, about half of the respondents have already used AI in clinical practice (15). Hence, we would like to encourage researchers, academics, and clinicians to continue conducting AI research in radiology and radiation oncology including safe and responsible use of AI and sharing their findings (16, 17). In this way, we can ensure continuous growth of our specialties and evidence-based practice for convincing more clinical centers to adopt AI and realizing its benefits in a wider context to improve patient outcomes.
